# Mortality Following Distal Femur Fractures Versus Proximal Femur Fractures in Elderly Population: The Impact of Best Practice Tariff

**DOI:** 10.7759/cureus.10744

**Published:** 2020-09-30

**Authors:** Islam Mubark, Amr Abouelela, Ahmed Genena, Abdallah Al Ghunimat, Islam Sarhan, Neil Ashwood

**Affiliations:** 1 Trauma and Orthopaedics, University Hospitals of Derby and Burton NHS Foundation Trust, Derby, GBR; 2 Trauma and Orthopaedics, James Paget University Hospitals NHS Foundation Trust, Norwich, GBR; 3 Trauma and Orthopaedics, Faculty of Medicine, Helwan University, Helwan, EGY; 4 Trauma and Orthopaedics, Faculty of Medicine, Alexandria University, Alexandria, EGY

**Keywords:** distal femur fracture, mortality rate, best practice tariff, elderly population, proximal femur fracture, hip, neck of femur fractures

## Abstract

Background and objectives

The mortality after hip, proximal femur, fractures in elderly patients has steadily declined in the last decade in the United Kingdom as a result of implementing of multiple protocols focusing on prompt multidisciplinary pre- and post-operative optimization and reducing time to surgery. The pinnacle of these protocols is the development of the best practice tariff as an incentive program for hospitals that meet set criteria by the National Health Service (NHS) England in managing these injuries. Until the time of writing this paper, there was no parallel program for the management of fractures involving distal femur in the elderly. The aim of this study is to evaluate the outcomes of distal femur fractures in elderly patients against proximal femur fractures regarding post-injury mortality, the prevalence of surgical treatment and time delay till surgery.

Methods

A retrospective study of all patients above the age of 60 admitted to Queens Hospital Burton between 2010 and 2014 with fractures involving distal end of the femur. Patient data were assessed for demographic criteria, co-morbidities as per Charleston Comorbidities Index, type of management, time-lapse before surgery and 30-day, six-month and one-year mortality. Results were compared to an age-matched control group of patients with proximal femur fractures randomly selected during the same time window.

Results

The main demographic criteria such as age, gender, and Charleston Comorbidities Index were similar in both groups. There were more patients treated non-operatively in the distal femur group than in the proximal femur group (15% vs 4%). Time to surgery was statistically significantly longer in distal femur group compared to the proximal femur (49.130 hours vs 34.075 hours, P = 0.041). The mortality in distal femur group was higher at all times (9.68% at 30 days, 20.32% at six months and 34.41% at one year) when compared to that in the proximal femur group (6.99% at 30 days, 14.52% at six months, 21.51% at one year).

Conclusion

The distal femoral fractures showed higher mortality at 30 days, six months and one year compared to the proximal femur group. This could be partly influenced by the implementation of best practice tariff in the proximal femur fracture group reflected in less time to surgery, pre- and post-operative multidisciplinary approach and more frequent operative management.

## Introduction

Hip fractures in the elderly have always been associated with a high rate of comorbidities and mortality with reported one-year mortality between 20% and 30% [1,2]. There is strong evidence that multidisciplinary approach in addition to reducing time to surgery is essential for reducing mortality in this frail patient group [3,4]. This has led the National Institute for Health and Care Excellence (NICE) in England to publish its guidance on hip fracture management emphasizing early surgery, pre- and post-operative optimization and multidisciplinary care [5]. In 2010, the National Health Service (NHS) improvement has implemented what is known as the best practice tariff. It is an incentive program that grants extra fund to hospitals that achieve certain targets in managing elderly patients with hip fracture. Amongst these targets are operating below 36 hours from the presentation and multidisciplinary approach including orthogeriatric review [6]. This approach has led to a significant reduction in 30-day-mortality in the United Kingdom in the last decade from 8.3% in 2009 to 6.1% in the year of 2018 according to the National Hip Fracture Database report 2019 [7].

Elderly patients sustaining distal femur fractures are usually frail and have multiple comorbidities. Mortality in this group of patients was reported being as high as in that following proximal femur fractures [8]. Until the time of conducting this study, the best practice tariff scheme only included proximal femur fractures which include intracapsular, intertrochanteric and subtrochanteric fractures, i.e. fractures within 5 cm distal to the lesser trochanter. There was no parallel best practice tariff for fracture femur sustained outside these anatomical areas. In this study, we assumed that elderly patients with distal femur fractures are similar in comorbidities to patients with proximal femur fractures. We also assumed that the lack of the best practice tariff may have led to delayed theatres and more frequent non-operative management, therefore higher mortality in the distal femur fracture group.

## Materials and methods

A retrospective study including all patients aged 60 years or more admitted to Queens Hospital Burton between 2010 and 2014. Inclusion criteria were closed fracture involving the lower third or distal end femur with or without extension to articular surface sustained following low energy trauma, i.e., simple fall from standing height. Exclusion criteria were pathologic, open and periprosthetic fractures.

Patients' records were reviewed for demographic criteria and comorbidities were scored using the Charleston Comorbidity Index (CCI).

An age-matched equal group of patients, admitted with proximal hip fractures (intracapsular, intertrochanteric and subtrochanteric), were randomly selected during the same time window as a control group.

Data were analyzed for the percentage of surgical treatment and time to surgery in each group. The primary outcome was mortality at 30 days, six months and one year.

## Results

Each group included 189 patients. The mean age for distal femur group was 81.1 years (range 60-99 ± standard deviation (SD) 8.1) and proximal femur was 81.8 years (range 61-101 ± SD 8.9). Females were the predominant gender in both groups with 65% and 69% prevalence in distal and proximal femur groups, respectively.

**Figure 1:**
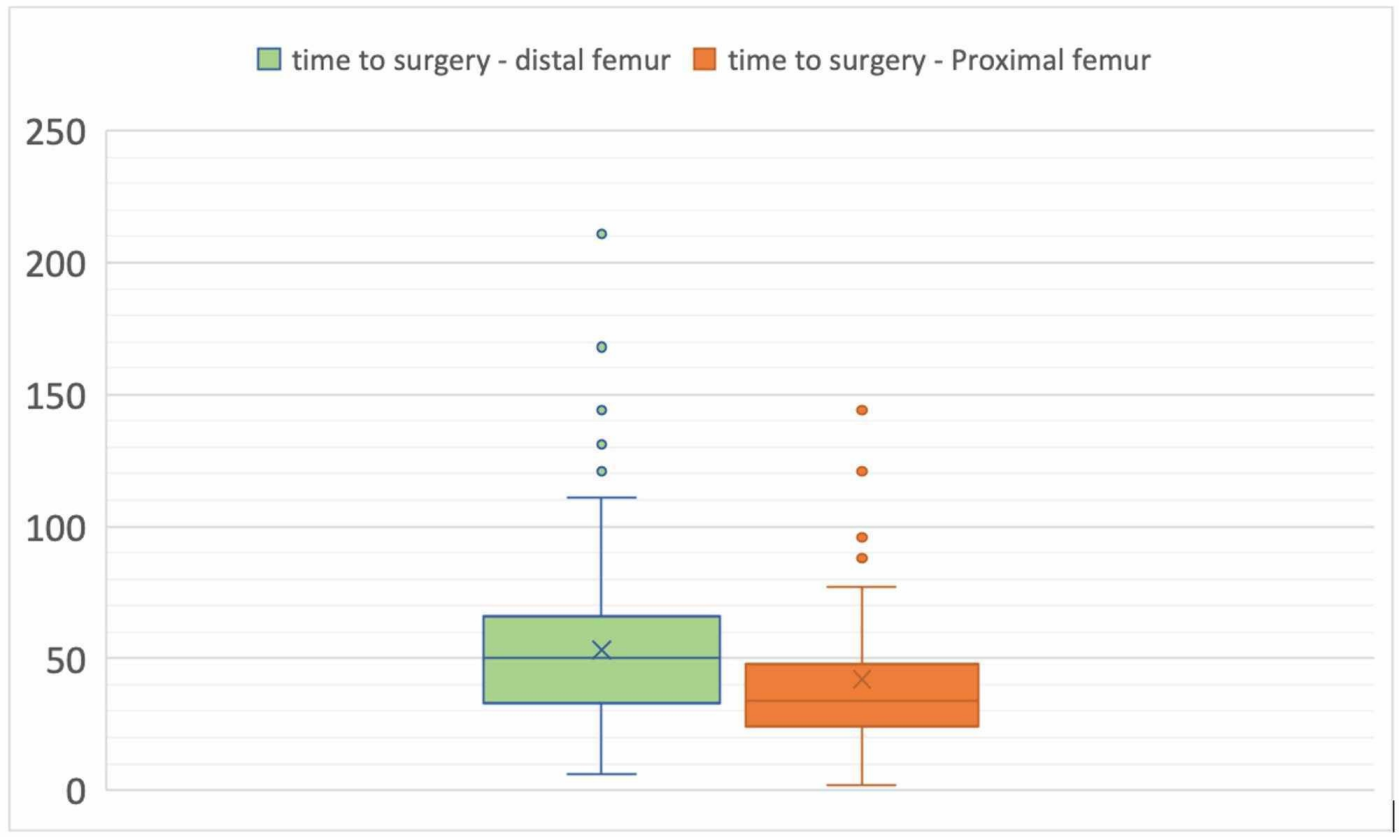
A box and whisker plot demonstrating the time to surgery in hours (Y-axis) in both fracture groups.

Statistical analysis was performed using Statistical Package for the Social Sciences (SPSS) version 23 (IBM Corp., Armonk, NY). There was no statistically significant difference in the mean CCI with distal femur mean CCI of (5.1 ± SD 2.1) against the mean of (4.8 ± SD 2.2) in proximal femur group (P = 0.612). The time-lapse till surgery in distal femur group (34.07 ± SD 2.9 hours) was statistically significantly longer than proximal femur group (49.13 ± SD 3.4 hours) (P = 0.041) (Figure 1) (Table 1).

**Table 1:** Difference in the two groups in terms of demographics, Charleston Comorbidity Index, time to surgery, percentage of surgery done within less than 36 hours and percentage of non-operative management.

	Distal femur fracture group	Proximal femur fracture group (control)	
Age in years Mean (range ± standard deviation)	81.1 (60-99 ± 8.1)	81.8 (61-101 ± 8.9)	
Females percentage	65%	69%	
Charleston Comorbidity Index (CCI) (mean ± standard deviation)	5.1 ± 2.1	4.8 ± 2.2	P = 0.612
Time to surgery in hours (mean ± standard deviation)	49.13 ± 3.4	34.07 ± 2.9	P = 0.041
Percentage of surgery done within less than 36 hours	46%	69%	
Percentage of non-operative management	15%	4%	

Only 46% of the distal femur group had surgery under 36 hours from admission versus 69% in the proximal femur group. There were more patients treated non-operatively in distal femur group (15%) compared to (4%) in the proximal femur group. The non-operative management was selected in distal femur group either as a result of undisplaced fracture which could be managed without operation in 25% or the patient was high risk for anaesthesia in the remaining 75%.

**Figure 2:**
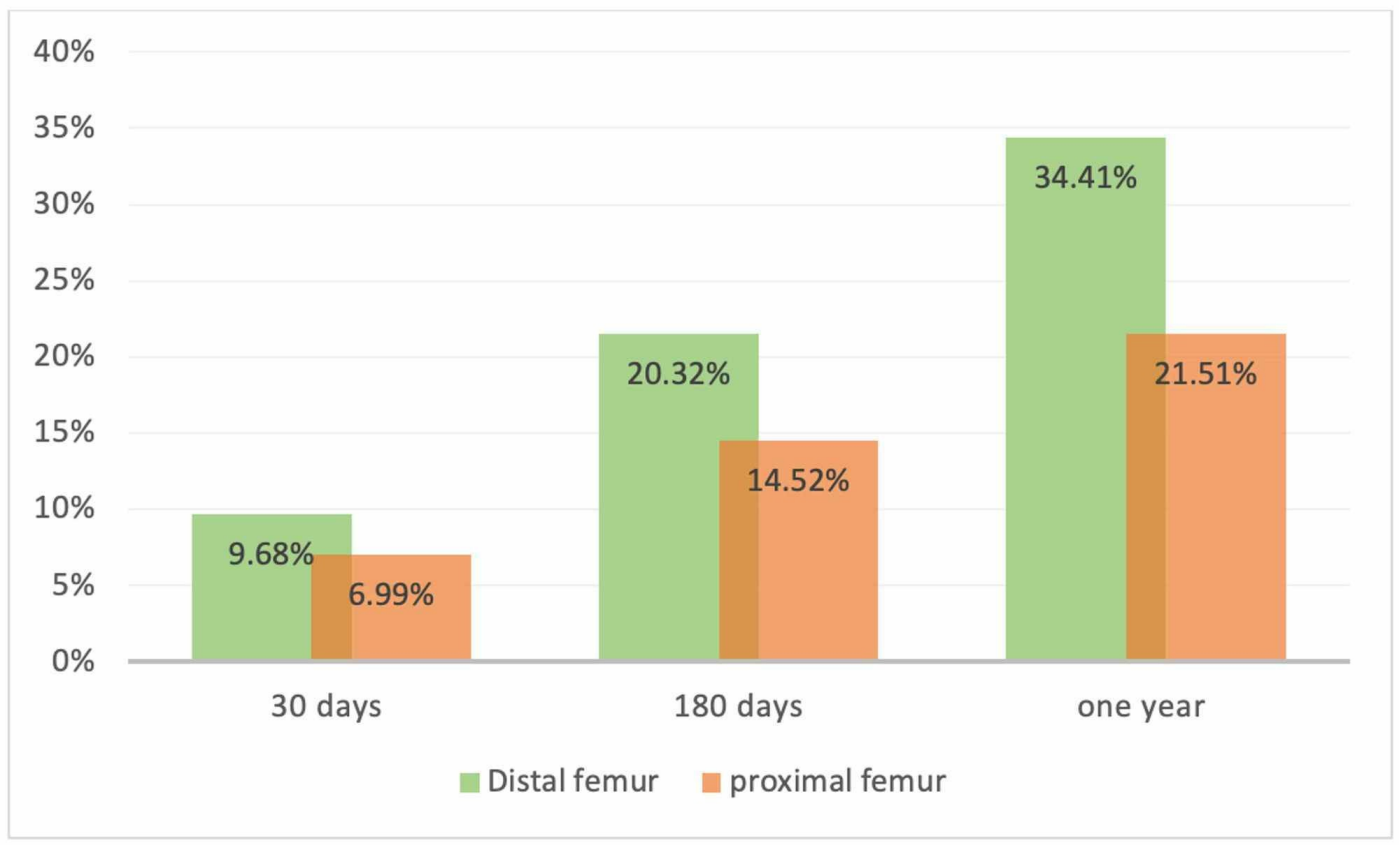
A bar chart demonstrating the mortality rate as a percentage (Y-axis) in both groups at 30 days, 180 days and one year (X-axis).

The distal femur group had higher mortality at all times (9.68% at 30 days, 20.32% at six months and 34.41% at one year), compared to the proximal femur (6.99% at 30 days, 14.52% at six months, 21.51% at one year) (Figure 2).

## Discussion

Hip fractures in the elderly have been associated with high morbidity and mortality owing to the prevalence of medical comorbidities in this cohort of patients with reports of 30-day mortality as high as 9.6%, while the one-year mortality rate increases to 33% [8,9]. There has been a large body of evidence that multidisciplinary care and operating within the first 48 hours after injury reduce the incidence of both morbidity and mortality [3,10]. In 2010, the National Health Service (NHS) in the UK has implemented what is known as the best practice tariff which provides additional funding to hospitals if they meet certain goals in managing these patients. These targets are (1) surgery within 36 hours of presentation, (2) assessment by a geriatrician within 72 hours of admission, (3) an abbreviated mental test performed before surgery, (4) a nutritional assessment during the admission, (5) a delirium assessment during admission and (6) a physiotherapist assessment on the day of or day following surgery [11]. This tariff was applied to patients aged 60 or older with fractures limited to intracapsular, intertrochanteric and subtrochanteric, i.e., within 5 cm below the lesser trochanter. This approach has led to a reduction in the UK reported 30-day-mortality in the last decade from 8.3% in 2009 to 6.1% in the most recent 2019 national hip fracture database report with almost 70% receiving surgery within 36 hours from admission in the emergency department and only 2.2% of patient treated non-operatively [7].

Distal femur fractures are the second most frequent fractures of the femur in the elderly population following those of the hip and their management is challenged by the presence of multiple medical comorbidities [12]. One study reported 11% of women with a distal femur fracture to have osteoporosis, 21% of patients have diabetes and more than 32% have a cardiovascular disease [13]. Mortality rates in the elderly with distal femur fractures have been reported to be around 18% at six months and 18-30% at one year [8,14,15].

In this study, we evaluated the outcome following fractures of the distal femur. The primary outcome was 30-day, six-month and one-year mortality against an age-matched control group of an equal number of proximal femur fractures' patients. The demographic criteria and comorbidities of both groups were comparable. We have been able to identify increased mortality at all times in the distal femur fracture group. This could be a combination of a higher percentage of time to surgery in this group in addition to more patients treated non-surgically. It is the authors' belief that the lack of best practice tariff cover for this group of patients could be a reason for these results and extending the best practice tariff to cover distal femur fractures could reduce the mortality in distal femur fracture close to proximal femur fracture. From April 2020, NHS England and NHS Improvement started the inclusion of distal femur fractures in the best practice tariff. It will be interesting to see how this would affect the mortality in this group of patients in the years to come.

## Conclusions

Elderly population sustaining distal femur fractures shared the same co-morbidities to those sustaining proximal femur fractures. Nevertheless, the distal femoral fractures showed higher mortality at all times. This could be partly attributed to the implementation of the best practice tariff reflected in less time to surgery, multidisciplinary pre- and post-operative approach and a higher percentage of operative treatment in the proximal femur fracture group.
